# PP70 Comparison Of Approval Dates Of New Substances Between The U.S. Food And Drug Administration And The European Medicines Agency

**DOI:** 10.1017/S026646232400240X

**Published:** 2025-01-07

**Authors:** Ariane Batscheider, Evgeniya Shlaen, Katharina Weiß, Darya Schnauffer, Roger-Axel Greiner, Peter Wagner, Doreen Bonduelle

## Abstract

**Introduction:**

Early access to innovative medications is a key factor for the quality of medical care. We investigated differences in approval dates of new active substances between the U.S. and Europe and a potential connection with company headquarter location.

**Methods:**

Data of new active substances (no generics, biosimilars, and hybrids) approved by the U.S. Food and Drug Administration (FDA) and the European Medicines Agency (EMA) from 1 January 2018 to 29 November 2023 were retrieved from FDA and EMA websites. We calculated the time difference between the drug approval dates between the agencies. We also retrieved the location of the headquarters of pharmaceutical companies.

**Results:**

Since 2018, 390 medicines were approved by FDA or EMA: 113 (29.0%) only by FDA versus 40 (10.3%) only by EMA, and 237 (60.8%) by both. Approval of 32.1 percent of medicines occurred within six months. Fifty-seven percent were approved more than six months earlier by FDA and 11.0 percent more than six months earlier by EMA. Overall, 45.6 percent of the headquarters are in Europe and 44.1 percent are in North America. For medicines approved by FDA only, 63.7 percent of headquarters are in North America and 26.5 percent are in Europe. This is reversed for medicines approved by EMA only. For substances approved by both, 50.6 percent of headquarters are in Europe and 39.2 percent are in North America.

**Conclusions:**

Almost 30 percent of new medicines approved by FDA within the last five years are not yet approved in Europe whereas only 10 percent are approved by EMA only. There is a tendency for companies with headquarters in North America to seek approval from FDA first. For medicines with approval from both authorities, most companies are primarily located in Europe.

